# In Silico Study of Anti-CD40 DNA Aptamers as Vaccine Adjuvants for Chickens

**DOI:** 10.3390/ijms27093808

**Published:** 2026-04-24

**Authors:** Juan Manuel Aceves-Hernández, Santiago Uribe Diaz, Abigeal Omolewu, Adil Sabr Al-Ogaili, Inkar Castellanos, María Inés Nicolas Vazquez, Alin Aurora Miramontes Salinas, Guillermo Tellez-Isaia, Young Min Kwon

**Affiliations:** 1 Chemistry Department, Facultad de Estudios Superiores Cuautitlán, Universidad Nacional Autónoma de México, Mexico City 04510, Mexico; nicovain@gmail.com (M.I.N.V.); aurizmiramon@gmail.com (A.A.M.S.); 2 Division of Agriculture, Department of Poultry Science, University of Arkansas System, Fayetteville, AR 72701, USA; su004@uark.edu (S.U.D.); alomolew@uark.edu (A.O.); icastell@uark.edu (I.C.); gtellez@uark.edu (G.T.-I.); 3 Laboratory Techniques, Department of Medical, Kut Institute of Technology, Middle Technical University, Baghdad 10074, Iraq; adil.saber@mtu.edu.iq

**Keywords:** chCD40, molecular docking, DNA aptamers, avian influenza, chickens, vaccines, adjuvants

## Abstract

We performed a protein-docking study for eight DNA aptamers (SEQ1–SEQ8) against chicken Cluster of Differentiation 40 (chCD40), which were experimentally identified via SELEX in our previous study. In silico and molecular docking analyses were performed to predict and obtain the secondary and tertiary structures of the aptamers. Aptamers SEQ3 and SEQ4, which showed the best inhibitory effects, were selected and utilized to produce a DNA-based vaccine adjuvant using rolling circle amplification (RCA). These aptamers had been previously characterized via mass spectroscopy to determine their molecular weight and regions that could potentially interact with chCD40. In the present study, these results were corroborated and expanded. A series of free software methods, including Mfold v.1.0, 3dADN v.2.0, ClusPro v.2.0, Hdock v.1.0, and PLIP v.1.0, were used to determine the aptamers’ secondary and tertiary structures and docking interactions, as well as the specific residues involved in the interactions and their distances. The structures were used to explain and thus understand their effect on the binding, selectivity, and stability of the aptamers. The main objective of the study was to determine whether these aptamers could be used as vaccine adjuvants against viral and bacterial pathogens, specifically chicken avian influenza. The docking results were in good agreement with the experimental and biological results. The procedure employed in this study could be an easy and effective tool for exploring the potential of the new technology of systematic evolution of ligands by exponential enrichment (SELEX) in the preparation of aptamers to control viral and bacterial infections as well as diseases, such as cancer and Alzheimer’s.

## 1. Introduction

The increasing attention on aptamer research has paved the way for the development of in silico approaches for the design, selection, characterization, and optimization of aptamers through advanced computational methods. Aptamers are short, single-stranded oligonucleotides (DNA or RNA) that form three-dimensional (3D) conformations, enabling them to bind to a multitude of targets, ranging from small molecules to complex structures. Given their unique characteristics, including a wide array of targets, high specificity and affinity, easy synthesis and modification, high reproducibility with low batch-to-batch variation, and good stability and selectivity, their application in diagnostics, therapeutics, and other research fields is being extensively explored [1].

Despite the growing interest in aptamer research, there remains a paucity of high-affinity aptamers for clinical applications. Aptamers are generally screened through systematic evolution of ligands by exponential enrichment (SELEX), an iterative process involving the evolution, purification, and enrichment of nucleic acids that bind to the target molecule with high specificity, selectivity, and affinity from a starting random library pool [2]. However, SELEX mechanisms are typically long, slow, and labor-intensive, requiring optimization to maximize aptamer binding affinity. To address these challenges, both analytical and in silico approaches have been introduced to improve the aptamer selection process. Notable analytical methods include combining SELEX with capillary electrophoresis [3], gel-based diffusion [4], or microarray technologies [5]. The integration of these platforms with in silico strategies has circumvented blind aptamer selection, shortened the screening time [1], and lowered the associated costs.

In silico strategies using various computational and simulation tools have significantly contributed to the advancement of aptamer screening and validation. Early work on aptamer design focused on minimizing the number of sequences in the starting library, which typically involves approximately 10^15^ random nucleic acid sequences. Preselection procedures using bioinformatics tools limit the sequences to those with high potential for target binding, ensuring that the candidate aptamers during each selection round have high binding affinity and selectivity for the target of interest [6,7]. With the numerous bioinformatic resources available, it is possible to predict the putative secondary and tertiary structures of aptamers and their targets, thereby revealing their thermodynamic properties. Simulations of aptamer–target complexes have facilitated virtual aptamer screening, the identification of structural motifs and key interaction residues, and the elucidation of the noncovalent interactions necessary for understanding aptamer–ligand affinity [8]. Insights from structural modeling have also enabled tailored chemical modification of aptamer sequences to improve their molecular recognition ability and stability [2,9,10].

Previously, using SELEX, we developed eight DNA aptamers with specific binding affinity and selectivity for the chicken CD40 extracellular domain (chCD40ED) [11]. These aptamers were used to design and develop rolling circle amplification (RCA) products to enhance binding affinity and immune responses. RCA is an isothermal process that produces long single-stranded DNA molecules with tandemly repeated sequences [12]. Each RCA was designed to alternatively present two aptamers as part of a long single-stranded DNA. Among the four RCA products evaluated (Aptamer RCAs I, II, III, and IV), Aptamer RCA I (which consists of SEQ1 and SEQ2) and Aptamer RCA II (which consists of SEQ3 and SEQ4), when conjugated with the M2e epitope peptide of the influenza virus, significantly stimulated anti-M2e IgG antibody production in chickens compared to M2e alone, demonstrating their potential as vaccine adjuvants (12). The aptamer RCAs mimic naturally occurring receptor–ligand interactions, leading to receptor oligomerization and activation of immune responses via the CD40 receptor on antigen-presenting cells (APCs) [13,14]. Our approach using RCA identified multiple anti-CD40 aptamers that could be used to enhance the efficacy of vaccines targeting various antigens beyond the M2e epitope, making it a versatile tool for both animal and human vaccine development.

Owing to the successful advancements in aptamer selection through in silico approaches, this study aimed to predict the secondary and tertiary structures and the binding mechanisms of eight candidate DNA aptamers from our previous work [11].

## 2. Results

### 2.1. Predicting the Protein Structure of Chicken CD40

Proteins are prominent targets in aptamer design and modeling due to their biological significance. CD40 is a member of the tumor necrosis factor (TNF) family which, together with its ligand CD40L, forms an immune-modulatory complex that serves as a target in vaccine and diagnostic development [14]. Despite its importance, structural information for CD40 in species other than humans is limited, with most available data focusing on CD40L [15].

The aptamers analyzed in this study were initially selected using chCD40ED as the target protein. In this study, the tertiary structure of chCD40 was generated using SWISS-MODEL through homology modeling. However, the resulting models demonstrated suboptimal quality, as indicated by low model scores. This limitation likely stems from the insufficient availability of protein structures in the Protein Data Bank (PDB), which serves as the primary repository for templates used in protein modeling, prediction, and analysis.

### 2.2. Secondary and Tertiary Structures of Potential Anti-chCD40 DNA Aptamers

In this study, the DNA sequences of the eight DNA aptamers described in our previous study [11] served as the starting point for constructing the corresponding secondary and tertiary structures.

The secondary structures of the most promising aptamers, SEQ3 and SEQ4, are illustrated in Figure S1. These structures include single-stranded segments at the 5′ and 3′ ends and a small hairpin stem-loop structure in the middle. The thermodynamic stability of each aptamer’s structure was assessed by calculating the Gibbs free energy (ΔG). The structures with the lowest ΔG values were identified as the most thermodynamically stable ones, indicating their suitability for further study.

The nucleotide number and type are presented in Table 1, along with the conformational energy of each of the eight aptamers. The different conformations are responsible for the characteristics of each aptamer, such as the amount and type of interactions between the protein and the ssDNA sequence. Thus, the affinity, selectivity, and bonding are also determined by the initial sequence conformation.

The corresponding secondary and tertiary structures of the eight aptamer sequences, with 40 and 80 nt, respectively, are shown in Figures S2 and S3 in the Supplementary Information. The chCD40 protein and eight aptamers were first docked using the ClusPro v.2.0 software (https://cluspro.org/login.php, accessed on 3 May 2024) [16] and then with the Hdock v.1.0 software, which uses a hybrid approach consisting of template-based modeling and ab initio free docking for protein–aptamer docking simulation [17].

The interactions were obtained from Molegro molexus.io/molegro-virtual-docker/, (accessed on 6 June 2024) and validated with Protein–Ligand Interaction Profiler (PLIP; https://plip-tool.biotec.tu-dresden.de/plip-web/plip/index, accessed on 15 May 2024) [18]. Chimera X 1.8, which provides tools for structure building and analysis, was used for visualization (https://www.cgl.ucsf.edu/chimerax/, accessed 3 June 2024) [16]. The results were obtained in PDB format using Gaussview (https://gaussian.com/gaussview6/, accessed 3 June 2024). The tertiary structures of the eight aptamers were obtained from biophy.hust.edu.cn/new/3dRNA/DNA (accessed 4 June 2024). Docking validation of the aptamers was performed using the Hdock software (http://hdock.phys.hust.edu.cn/) [19], and Pymol was used for visualization.

### 2.3. Noncovalent Interactions Between Aptamers and Protein Receptors

To determine the influence of the type of nucleotide on the secondary structural conformation of each aptamer sequence, the free energy ΔG (kcal/mol) for each sequence was calculated and is presented in Table 1, with the corresponding amount of each type of nucleotide. Noncovalent interactions between aptamers as ligands and proteins as receptors were analyzed using the Protein–Ligand Interaction Profiler (PLIP) web server (Table 2 and Tables S3 and S4). Among the evaluated protein–aptamer complexes, the SEQ4–chCD40 complex exhibited the highest number of interactions, which included hydrophobic interactions, salt bridges, π-stacking, and hydrogen bonds, totaling 24 interactions. In comparison, the SEQ3–chCD40 complex displayed 38 interactions. Based on these results, the two aptamer–protein complexes with the highest number of interactions were selected for further evaluation. It should be noted that aptamers SEQ3 and SEQ4 have the highest number of G nucleotides and were the best inhibitors in the wet experiments. Interestingly, aptamers SEQ3 and SEQ4 presented the highest docking energy values despite having lower amounts of the amino acid cysteine and the highest amount of the nucleotide guanidine (16 G).

Analysis of the specific amino acids involved in these interactions revealed that arginine (Arg) was the most involved, contributing to 23.1% of the total interactions across all model complexes, followed by threonine (Thr; 15.4%), lysine (Lys; 12.87%), and histidine (His; 9.48%) (Table 2). Interestingly, the positively charged residues Arg and His were only involved in 23.1% and 9.48% of the total interactions, respectively, probing the conventional expectation of strong binding between negatively charged aptamers and positively charged amino acid residues. Furthermore, threonine and arginine were predominantly involved in hydrophobic interactions.

The high affinity and selectivity values suggest that these aptamers adopt stable structural motifs, with high attraction energy and orientation towards special amino acid residues. The predicted tertiary structures generated using 3dRNA showed shared patterns, primarily differing in the orientation of the hairpin loops. This folding pattern reflects the influence of nucleotide composition and experimental conditions, such as ionic strength and temperature, on structure determination. The tertiary structures with the lowest energy scores were selected for molecular modeling of the aptamer–protein complexes. However, further experiments were required to validate the specific binding interactions with cell surface proteins, especially chCD40, in order to confirm the aptamers’ selectivity and eliminate their cross-reactivity with these proteins. This study provides an exploratory simulation of potential interactions between aptamers and the chCD40 target protein; its limitations are acknowledged and will be addressed in future research.

### 2.4. Aptamer and ChCD40 Protein Complexes

**Figure 1:**
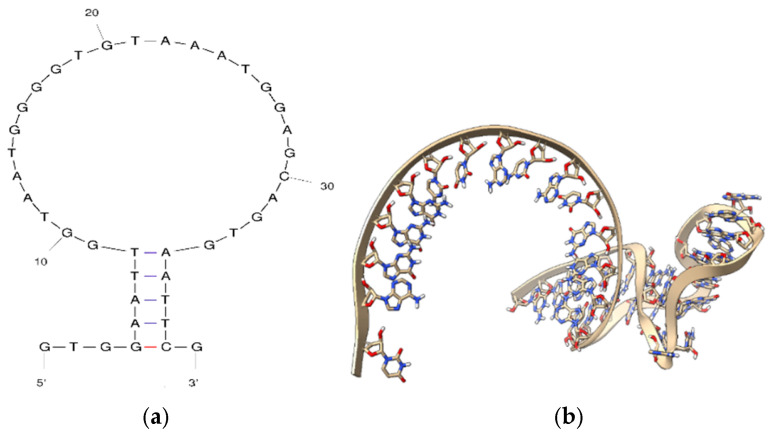
(**a**) Secondary and (**b**) tertiary structures of SEQ3 (conformational energy: ΔG = −0.65 kcal/mol) obtained from Mfold. The aptamer sequences are from our previous study [11,15].

Molecular docking simulations were performed and validated using HDOCK, with the tertiary aptamer structures and protein templates used as inputs. The goal was to evaluate the interactions between each aptamer and chCD40. These studies aimed to understand how the properties of the target protein determine the affinity, selectivity, and stability of the aptamers. Secondary and tertiary structures of SEQ3 obtained from Mfold are in Figure 1.

The docking study first examined the interactions between chCD40 and each aptamer sequence. The best model for each aptamer–protein pair was selected from the generated docking models based on the scoring function, which evaluates docking quality. Models with the lowest docking scores were chosen to identify the binding mechanisms and interactions. Tables S1 and S2 present the parameters of the docking scores.

### 2.5. Negative Control Aptamer

**Figure 2:**
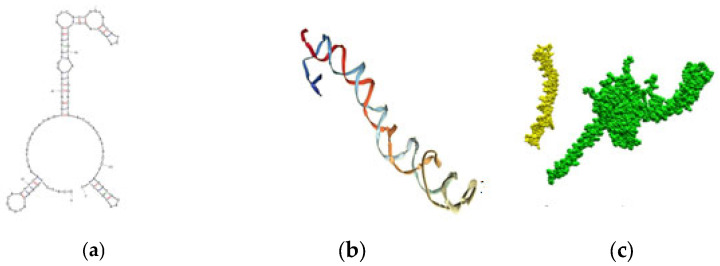
Negative control’s (**a**) secondary structure and (**b**) 3D structure. (**c**) No interactions and no docking of NC (yellow) with the chCD40 (green).

The Random DNA Sequence generator can generate a random sequence of a specified length that can be used to evaluate the importance and significance of sequence analysis results. We experimentally tested a random sequence and found that it did not show affinity, selectivity, potency, or reactivity. This sequence served as the negative control (NC). The same docking methodology was applied to the NC aptamer. The corresponding secondary and 3D structures of the NC, and its docking and interaction results are shown in Figure 2.

In the NC aptamer–chCD40 docking study, there were no interactions, Figure 2c. These results validate our docking method, since the experimental results also showed that NC had no affinity, selectivity, nor inhibitory effect. The simulation methodology also produced similar results to the experimental findings for interactions with the positive control.

**Figure 3:**
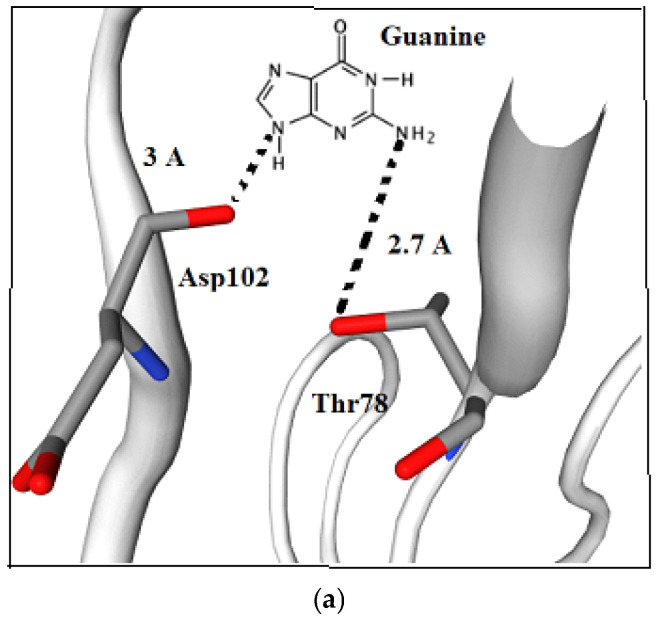

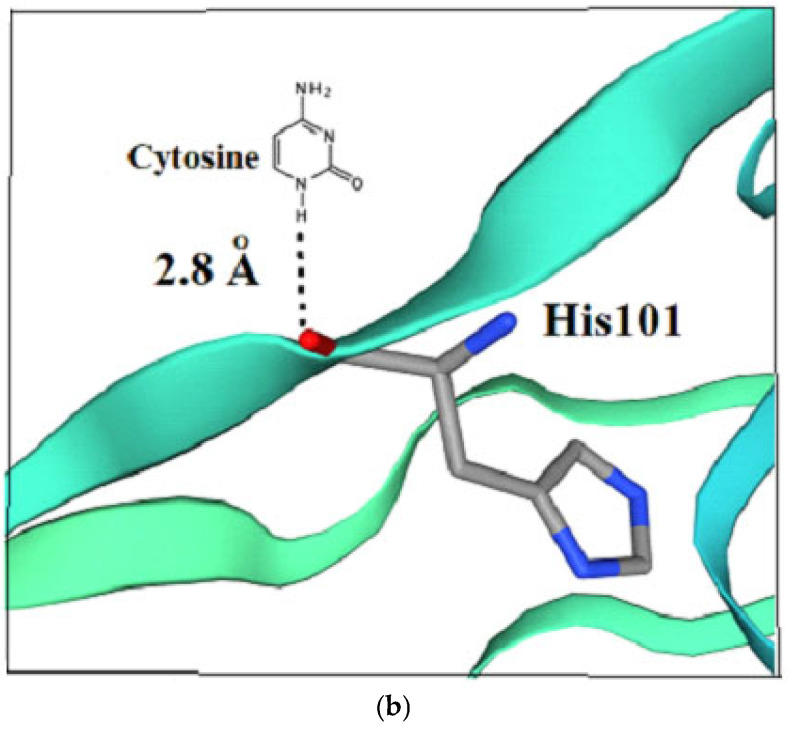
(**a**) Aptamer SEQ3–chCD40 complex after docking, residues Asp102 and Thr78 interacting with guanine of aptamer SEQ3 at distances of 3.0 and 2.7 A, respectively; H bonding shown as dotted line. (**b**) Aptamer SEQ4–Cd40 complex after docking, residue His101 of CD40 interacting with cytosine of aptamer SEQ4 at distance of 2.8 A. Oxygen atoms are in red color, nitrogen is in blue and carbons are in gray.

Representative docking models (aptamers SEQ3 and SEQ4 with chCD40) are shown in Figure 3, where hydrogen bonds are highlighted in blue. These findings provide insights into the molecular interactions governing aptamer–protein binding and offer a foundation for further experimental validation.

The selectivity, affinity, and stability of the aptamers can be attributed to intramolecular nucleotide base pairing, which enables the molecule to fold into specific conformations [20]. Mfold predictions rely heavily on the minimum free energy (ΔG) as the core metric, along with the base pair interaction energies. The conformation with the lowest ΔG value is selected as the most thermodynamically stable structure [16,19]. Environmental factors such as temperature and ionic concentrations, which affect aptamer folding, are incorporated into Mfold’s parameters to mimic in vivo conditions [19]. However, accurately replicating the complex SELEX conditions remains a challenge in in silico molecular docking of aptamers with target proteins.

The binding, affinity, and specificity of DNA aptamers are closely tied to their unique tertiary structures. Aptamers adopt complex folds comprising hairpins, loops, pseudoknots, and G-quadruplexes, which enable high-affinity binding to target protein surface epitopes [15]. These structural variations stem from the flexibility of their phosphodiester backbone, which allows for a wide range of torsional angles and generates diverse tertiary structures. Consequently, aptamers exist in multiple conformations in solution, each with distinct binding affinities.

Computational tools for tertiary structure prediction of DNA aptamers are currently limited and are often adapted from RNA-focused applications. It was demonstrated that in silico structural conversion between DNA and RNA aptamers yields similar hairpin conformations (21). At present, by substituting thymine with uracil in the sequence and using tools such as 3dRNA, the tertiary structure of DNA aptamers can be modeled. The final DNA structure is obtained by reverting the sugar residues (from 2′-OH to 2′-H), bases (uracil to thymine), and backbone (ribose to deoxyribose).

**Figure 4:**
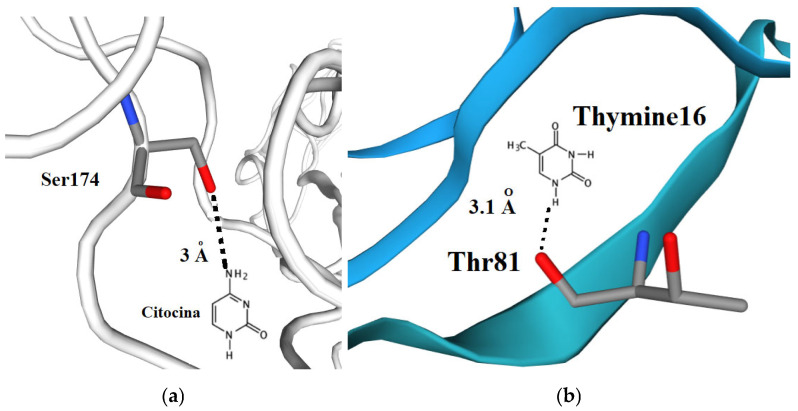
(**a**) Aptamer SEQ4–chCD40 complex. Residue Ser174 of CD40 interacting with aptamer SEQ40 at distance of 1.0 Å. (**b**) Residue Thr81 of CD40 protein interacting with Thymine16 of the aptamer SEQ4 at distance of 3.1 Å. Oxygen atoms are in red, nitrogen in blue and carbon in gray.

Following determination of the aptamer tertiary structures, molecular docking was performed to predict aptamer–protein interactions based on the lowest ΔG docking scores [6]. Docking algorithms typically fall into two categories: template-based and machine learning approaches [9]. The Hock server employs a hybrid strategy, combining template-based modeling and ab initio docking. This flexibility enables the server to process docking based solely on the input sequence and structural information, even in cases where experimental structures are unavailable. Figure 4 shows the tertiary structure and interactions of the aptamer SEQ4–chCD40 complex after the docking process.

Although the docking results are approximations of potential interactions, they provide insights into aptamer binding sites and structural motifs. Aptamers bind to protein targets through various interactions, including hydrogen bonding, hydrophobic interactions, electrostatic interactions, salt bridges, van der Waals forces, and π-stacking [16]. Surface protein epitopes typically feature electropositive and polar regions dominated by hydrogen bonds and charge–charge interactions, while hydrophobic interactions play a limited role in nucleic acid binding [21].

Hydrophobic interactions, which are pivotal in protein–aptamer interactions, can be enhanced through pre- and post-SELEX chemical modifications to the aptamers. However, in this study, the native 3D structures were used, which revealed predominantly hydrogen bonding and limited hydrophobic interactions with protein side chains. Hydrogen bonds, the most common interaction type, involve amino acid side chains (which act as donors) and the negatively charged phosphate backbone of the aptamers (which acts as an acceptor) [16]. These interactions stabilize the aptamer–protein complex, particularly through polar amino acids like serine, threonine, and glutamine [15,19,21,22,23].

Conversely, hydrophobic interactions occur at nonpolar residues and are commonly observed in engineered aptamers with functional groups designed to enhance binding affinity [20,24,25]. Docking algorithms allow for identification of the best aptamer–protein complexes based on predicted tertiary structures and docking scores. In our work, aptamers SEQ3 and SEQ4 exhibited the highest number of interactions (38 and 24, respectively), which included hydrogen bonds and salt bridges (Table 2). Interestingly, aptamer SEQ4 achieved the highest docking score (−398.45 A.U.), surpassing the scores of aptamer SEQ3 (−346.78 A.U.). However, the docking scores alone did not directly correlate with the experimental selectivity and affinity values, as the type of charge and polarity of the amino acid residues are also very important. This highlights the need for further validation of the computational predictions.

### 2.6. Negative Control Protein

In addition, to validate the results for the protein target (CD40), similar proteins, including 6PE8, 4GIQ, and Alpha fold model AoA803Y327.1, were docked to aptamers SEQ3 and SEQ4. The percent identity of these proteins compared with CD40 was 56.8%, 64.4% and 92.2%, respectively. The results of the docking simulation found only two, three, and five H bonds, respectively. These results validated that aptamers SEQ3 and SEQ4 have higher selectivity and specificity for CD40 (Table S3).

The assay results for the eight aptamers demonstrate their capacity to detect and interact effectively with the target protein, highlighting their potential for therapeutic development. Nevertheless, additional experimental validation is required to confirm these preliminary findings. Beyond sensitivity, specificity remains a critical parameter in defining the performance and safety of a therapeutic [15]. Comprehensive specificity evaluations against chCD40 are essential to minimize cross-reactivity with other pathogens and to ensure that these aptamers do not face interference or competition from endogenous host ligands. In silico aptamer maturation offers a robust framework for maximizing the binding capacity toward target ligands while minimizing off-target interactions with host proteins. Given that the intricate binding interface between aptamers and ligands is often difficult to fully characterize via in vitro experiments alone, a complementary computational approach is essential for both streamlined screening and a deeper understanding of nucleic acid–protein dynamics. This combinatorial strategy—integrating in silico design with in vitro SELEX—has already been proven effective in developing potent therapeutic aptamers; for example, for thrombin [20,25,26] and the SARS-CoV-2 spike protein [27,28]. Furthermore, our current workflow facilitates the simulation of aptamer binding across diverse matrices and structurally related compounds, allowing for targeted modifications that reduce cross-reactivity. Despite the inherent limitations of this study, it demonstrates that a bioinformatics-driven approach remains central to the evolution of next-generation aptamer therapeutics.

The interactions from the docking study of the chCD40–aptamer SEQ complex were analyzed by inputting the docking results from the Hdock v.1.1 program into the PLIP v.1.0, software to analyze the different interactions, including H bonding, hydrophobic interactions, Π interactions, and salt bridges. Salt bridges are mainly due to phosphate ions, which play an important role in aptamer interactions with target proteins.

### 2.7. Types of Interactions in Aptamer SEQ–Protein Complexes

The positively charged amino acids Arg, His, Lys, Asp, and Glu were all involved in the interactions between the eight aptamers and chCD40 protein; aptamers SEQ3 and SEQ4 were selected for further RCA aptamer preparation, as reported previously [11].

It is clear that aptamers SEQ3 and SEQ4 had the highest number of interactions (38 and 24, respectively; (Table 2), indicating their superior affinity, selectivity, and specificity. The PLIP results indicated that the same residue, His69A, interacts with different donor atoms, such as the O3 or O2 atoms of aptamer SEQ3 skeleton, probably the phosphate. Thus, when analyzing a specific interaction, the interaction with the shortest distance and/or more negative free energy of interaction (ΔG) should be used.

Hydrophobic interactions are defined in the PLIP software as interactions between carbon atoms of the ligand and atoms other than H of the receptor; these atoms can also be involved in other types of interactions. It is noteworthy that only a few of the 20 amino acid residues in chCD40 interact with all the aptamer sequences. One of these amino acids is Arg, which was involved in interactions with all aptamers except for aptamer 8, which had the lowest affinity, selectivity, and potency. The other residues were His and Lys, which interacted with all eight aptamer sequences. Aptamers SEQ3 and SEQ4 had higher numbers of this amino acid, as well as other residues with a minor presence, such as Glu, Gln, and Arg; these aptamers had the highest affinity, selectivity, and potency. Aptamer SEQ3 also has Tyr and Thr residues, while aptamer EQ4 has Ser residues. It is important to mention that the amino acids’ characteristics, such as polarity or basic or acidic nature, influence the aptamers’ properties and their potential applications as adjuvants.

The amino acids Gln, Tyr, and Ser only showed interactions with five aptamers. Therefore, it is possible that some amino acid residues are important in some types of interactions and for desirable properties for the aptamers, which affect their therapeutic potential, for example, as adjuvants in chicken vaccines.

There was a total of 180 interactions between the eight aptamer sequences and chCD40 (Table 2). The percentages of positively charged amino acids involved in these interactions were as follows: 23.9% Arg, 16.6% Hist, 12.2% Lys, and 5.56% Glu. The percentages of negatively charged amino acids were 8.33% Gln, 3.33% Asn, 3.88% Thr, and 1.6% Ser, which only accounted for 17.14% of the total interactions. However, it is worth mentioning that other charges came from the phosphate skeleton of the ssDNA, some of which were involved in salt bridge interactions. The hydrophobic amino acids Ala, Met, Phe, Tyr, and Trp only participated in 10.56% of the interactions. As special cases, Cys and Pro only participated in 1.2%. Since there are few reports of ssDNA aptamers for proteins, it is important to validate the theoretical results using X-ray crystallography and RMN.

**Figure 5:**
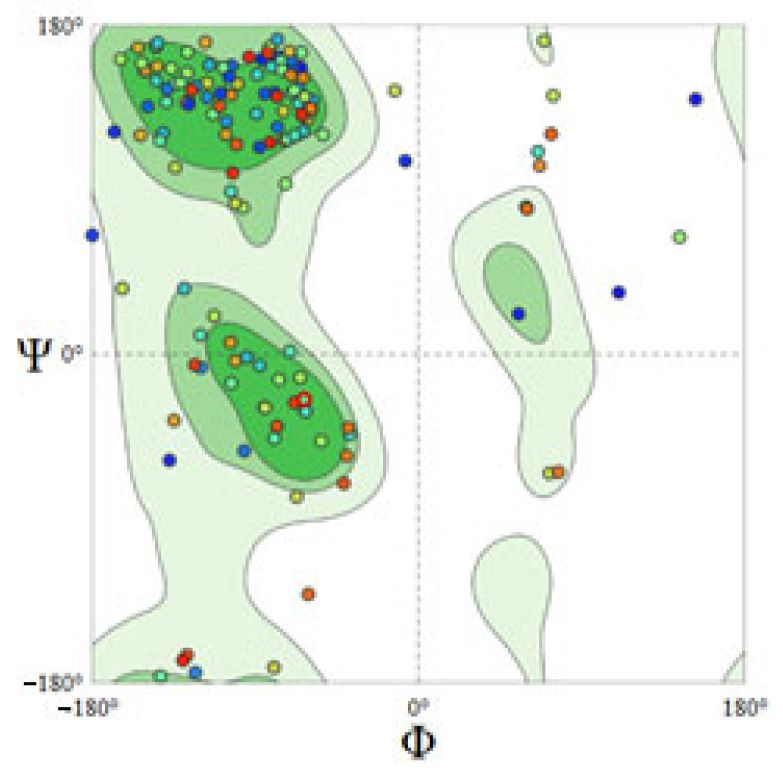
Ramachandran plot of protein target chCD40.

Interestingly, two random sequences were obtained using the Sequence Manipulation Suite. Each sequence is 80 nt long and was used to validate our docking results. The chCD40 protein was used in a docking study with the random sequences. As expected, no significant docking results were obtained: the RMSD values were large (>200 A) and only one weak interaction was obtained from the docking simulation with random sequence NC2. Furthermore, the only interaction was with an amino acid residue outside the inhibitory area of chCD40. Thus, these and the NC aptamer results validated the docking method, which found that aptamers SEQ3 and SEQ4 had good affinity, selectivity, and reactivity, in agreement with the experimental results. In addition, three CD40 similar proteins were tested as negative controls: proteins PDB id:6PE8 and 4GIQ, and AlphaFold model AOA803y327, with 56.8%, 64.4%, and 92.2% identity, respectively. Only two, three, and five weak H bonds were detected in the molecular docking simulation. The docking scores are shown in Table S2. Our experimental and docking results showed good agreement. We plotted Ramachandran plots to verify that the torsion angles and folding are correct. The CD40 Ramachandran plot is presented in Figure 5, which shows that all but a few of the residues are located in the correct position.

Aptamer maturation can also be performed in silico to maximize its binding capacity to target ligands and eliminate the possibility of binding to host proteins. Interactions between aptamers and their ligands are complex and difficult to evaluate using in vitro experiments alone; thus, complementary in silico aptamer design can be carried out to facilitate the screening of aptamers and further our understanding of nucleic acid–protein interactions. Combinatorial in silico aptamer design, in conjunction with in vitro SELEX experiments, proved to be effective in designing aptamers for thrombin [20,25,26] and SARS-CoV-2 spike protein detection [27,28], which could be developed into potent therapeutic tools. Furthermore, our workflow permits the simulation of aptamer binding to different matrices and other structurally related compounds; thus, efficient modification can be employed to limit cross-reactivity. Despite the limitations of this study, it demonstrated that employing a bioinformatics approach is still central to the development of aptamer-based therapeutic technologies. A detailed analysis was difficult to perform as there were more than 3500 heavy atoms, which are different from H atoms. Therefore, only the influence of AA clusters could be observed; however, these interactions may differ from those involving small-molecule ligands [29,30].

## 3. Materials and Methods

### 3.1. Sequences, Aptamers, and Interaction with Exterior Membrane Protein Targets

This study focused on the development and characterization of DNA aptamers with high binding affinity for the extracellular domain of chicken CD40 (chCD40ED). Initially, a panel of eight DNA aptamers was generated via cell-SELEX by a third-party laboratory, with candidates selected based on their enrichment frequency and preliminary binding profiles. The primary objective of the current study was to elucidate the specific binding sites of these aptamers within the target protein architecture. We demonstrated that all eight candidate aptamers significantly induced signal transduction in the chicken HD11 macrophage cell line (*p* < 0.05). Notably, the RCA II aptamer (comprising aptamers SEQ3 and SEQ4) was conjugated to the M2e epitope of the influenza virus as a model hapten for in vivo evaluation.

Following immunization in chickens, the RCA II–M2e complex elicited significantly higher titers of anti-M2e IgG antibodies compared to the M2e epitope alone (*p* < 0.05). These results suggest that the RCA II aptamer could serve as a potent molecular platform for enhancing vaccine efficacy, offering broad potential for targeting diverse antigens beyond the M2e model used here.

**Figure 6:**
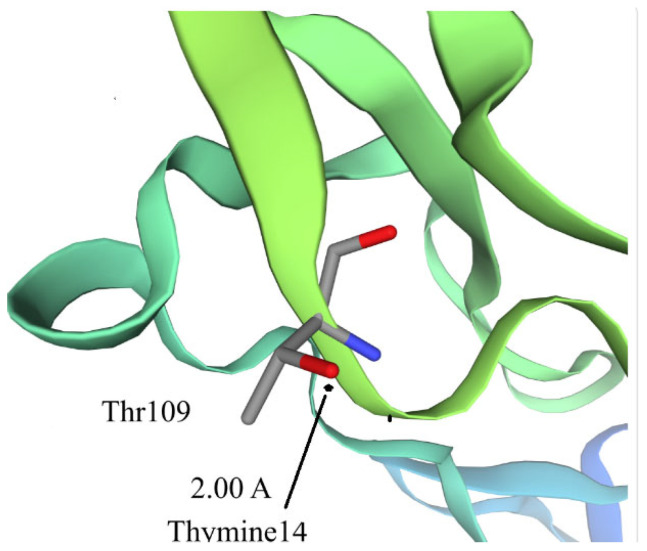
Interaction between protein CD40’s Thr109 with SEQ3 aptamer’s Thymine14. Oxigen atom in red, nitrogen atom in blue and carbon atoms are in gray.

To further evaluate the aptamers’ binding properties, LC–ESI–mass spectrometry was performed to identify the binding sites of chCD40ED protected by DNA aptamers SEQ3 and SEQ4. Limited proteolysis experiments under physiological conditions, followed by LC–ESI–MS analysis, revealed solvent-accessible lysine and arginine residues. When chCD40ED formed a complex with aptamer SEQ3 or SEQ4, these residues became less accessible to trypsin digestion, resulting in lower LC–ESI–MS signal intensities compared to the control without aptamer binding. Aptamers SEQ3 and SEQ4 provided consistent and significant protection, highlighting their strong binding interactions with chCD40ED and motivating further study and analysis of these aptamer–protein interactions. Interaction between protein CD40’s Thr109 with SEQ3 aptamer’s Thymine14 is presented in Figure 6. 

### 3.2. Software Workflow

**Figure 7:**
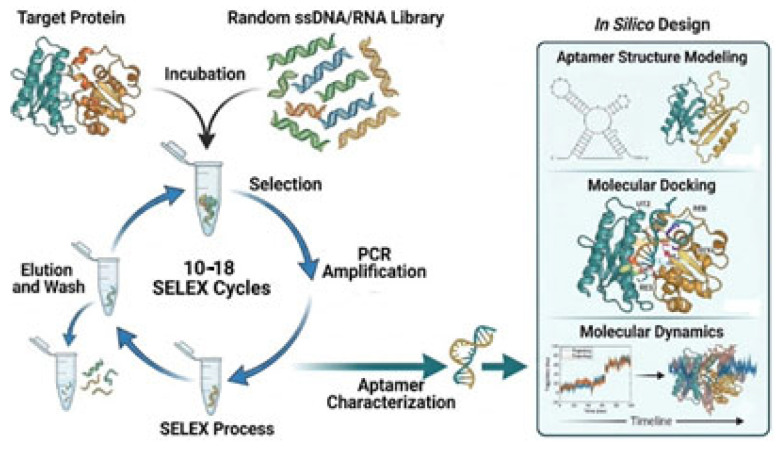
Workflow for aptamer characterization after SELEX screening. Different colors represent different proteins or different sequences.

The bioinformatics workflow based on free tools was validated using random sequences [12] to predict the binding residues involved in aptamer–target protein interactions. The DNA aptamer nucleotide sequences were first used to predict their secondary structures using the Mfold web server (http://www.unafold.org/mfold/applications/dna-folding-form.php, accessed on 19 September 2024) [16]. Workflow for aptamer characterization is presented in Figure 7.

The default parameters were used, with ionic conditions set as [Na+] = 1.0 M and [Mg2+] = 0.0 M at 37 °C. For each aptamer, the most thermodynamically stable structure—characterized by the lowest Gibbs free energy—was selected, and the corresponding Vienna file (dot-bracket format [.b] file) was saved [15]. Protein–aptamer interactions were obtained using the Protein–Ligand Interaction Profiler program (PLIP) (https://plip-tool.biotec.tu-dresden.de/plip-web/plip/index, v.1.0; accessed 20 July 2024).

The amino acid sequences of the selected target protein, including chCD40, were obtained from UniProt v.2.0, (https://www.uniprot.org/, accessed 5 April 2024). These target proteins, which play critical roles in the pathogenicity of the microorganism in chickens, were selected due to their relevance in vaccine and diagnostic research. 

## 4. Conclusions

A comprehensive bioinformatics and in silico pipeline were employed to characterize the molecular interactions between the candidate aptamers and the chCD40 surface protein. Based on their critical involvement in binding, aptamers SEQ3 and SEQ4 were used as the primary components for the development of the RCA II-SA-M2e complex, which elicited significant anti-M2e IgG titers. Molecular docking of these aptamer–protein complexes effectively demonstrated the anchoring of the sequences to the protein surface and identified the key residues essential for structural stability and selectivity. Furthermore, the docking results corroborated the interaction interfaces previously predicted based on mass spectrometry analysis. To build upon these findings, future studies should quantitatively evaluate the aptamers’ binding affinities and selectivity, as well as exploring the integration of pre- or post-SELEX modifications to further optimize their binding capacity.

In addition, other methods could be used when more secondary and 3D structures of ssDNA aptamers are added to relevant databases to increase the number of structures available for comparison and evaluation. The negative control (NC) gave similar results when evaluated using the experimental and theoretical methods, validating our methodology. Despite its limitations, the in silico approach is still a powerful method for expanding our knowledge on DNA aptamer–protein interactions and represents a promising strategy for the development of aptamer technology, uses, and applications. In this study, there was a good agreement between the experimental and theoretical results in terms of interactions and the binding and selectivity of the aptamers. However, further in silico studies are needed to optimize the aptamers in terms of ssDNA nucleotide number and sequence, in order to obtain better inhibitory properties.

Molecular docking results presented herein represent the most probable structural models of the aptamer–protein complexes and are primarily used to rationalize and interpret the corresponding experimental findings. Nevertheless, it is important to acknowledge that molecular docking approaches possess inherent limitations, and thus only moderate conclusions can be drawn regarding the precise nature of intermolecular interactions and binding affinities. Further validation and justification of these results can be achieved through molecular dynamics simulations, which provide a more accurate and dynamic representation of complex stability, conformational flexibility, and thermodynamic properties. Accordingly, ongoing work in our group is focused on implementing molecular dynamics methodologies to strengthen and validate the findings of the present study, as well as to support future investigations involving aptamer-based systems targeting viral pathogens, cancer-related biomarkers, Alzheimer’s disease, and other emerging diseases.

## Figures and Tables

**Table 1:** Number and type of nucleotides in each sequence, and secondary structure conformation energy (in kcal/mol).

Aptamer	Nucleotide Amount and Type	Secondary Structure Conformation Energy, ΔG (kcal/mol)
1	16A 9C 10G 5T	−1.24
2	14A 11C 11G 4T	−2.78
3	11A 2C 16G 11T	0.70
4	13A 5C 16G 6T	−0.71
5	5A 8C 9G 16T	−0.72
6	9A 9C 10G 10T	−1.03
7	9A 6C 13G 12T	−1.04
8	9A 16C G 14T	1.66

**Table 2:** Interactions between aptamers and chCD40 protein.

Aptamer	Hydrophobic Interaction(s)	Hydrogen Bonding	Π Stacking	Salt Bridge(s)	Total Number of Interactions
1	Tyr	5Asn, Asp, 3Arg, Cys, 2Gln, 3His, Lys, Ser, 3Thr	His	Asp	**23**
2	Ala	Asn, Asp, 3Gln, Glu, 2Lys, Thr, Tyr	His, Lys, Arg, Glu	Phe	**16**
3	Arg, Thr	5Arg, Gln, 3Glu, 7His, Lys, 2Ser 2Tyr	Arg	3Arg, 4Glu, 5His, 2Lys	**38**
4	0	2Arg, 2Gln, Glu, 2His, Lys, Ser	His	5Lys, 5Arg, 2His, 2Glu	**24**
5	Tyr, Met	6Arg, 2Tyr, Lys, Trp, His	0	3Arg, His	**17**
6	0	2Arg, 2Gln, Glu, 2His, Lys, Ser	His	5Lys, 5Arg, 2His, Glu	**23**
7	Tyr, Met	Ala, 4Arg, His, Lys, 2Trp, 2Tyr	0	3Arg, His	**17**
8	Pro, Thr, Trp	2Asn, 5Gln, 4His, Thr, Tyr	His	2Lys, Glu	**20**

## Data Availability

The original contributions presented in this study are included in the article/Supplementary Materials. Further inquiries can be directed to the corresponding authors.

## Supplementary Materials

The following supporting information can be downloaded at: https://www.mdpi.com/article/10.3390/ijms27093808/s1.
