# Supplementary material for: In Silico Study of Anti-CD40 DNA Aptamers as Vaccine Adjuvants for Chickens
Source: Int J Mol Sci. 2026 Apr 24;27(9):3808. doi: 10.3390/ijms27093808 (PMC13163442; doi:10.3390/ijms27093808)
Supplement: Supplementary file 1 [file ijms-27-03808-s001.zip › ijms-4143578-supplementary.pdf]

Supplementary File

Supplementary Information

The properties and characteristics of the Aptamers are determined by the number and sequence of the nucleotides, A, G, C, and T. In the presented work, 8 aptamers with 40 nucleotides were obtained.

The secondary structures of the main sequences are in Figure S1.

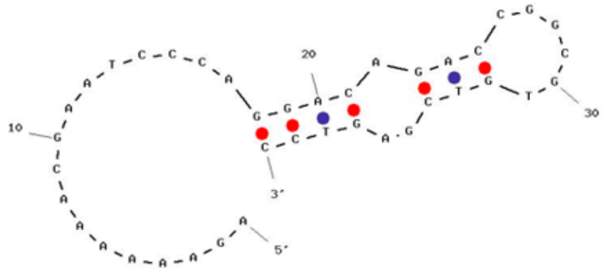

a)

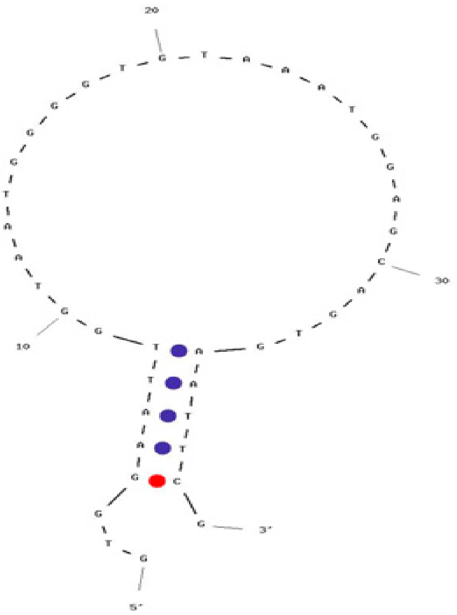

b)

Figure S1. Secondary structure of Sequences of 40 nt. (a), AptamerSeq3, (b) AptamerSeq4, by using Mfold software.

Results of the docking study and the interactions analysis show that aptamers conformation are very important in their affinity, stability and selectivity. It seems that H bonding is the crucial interaction determining the eficacy of the inhibitory effect of the aptamers. The tertiary structure of the 8 aptamers are presented in Figure S2.

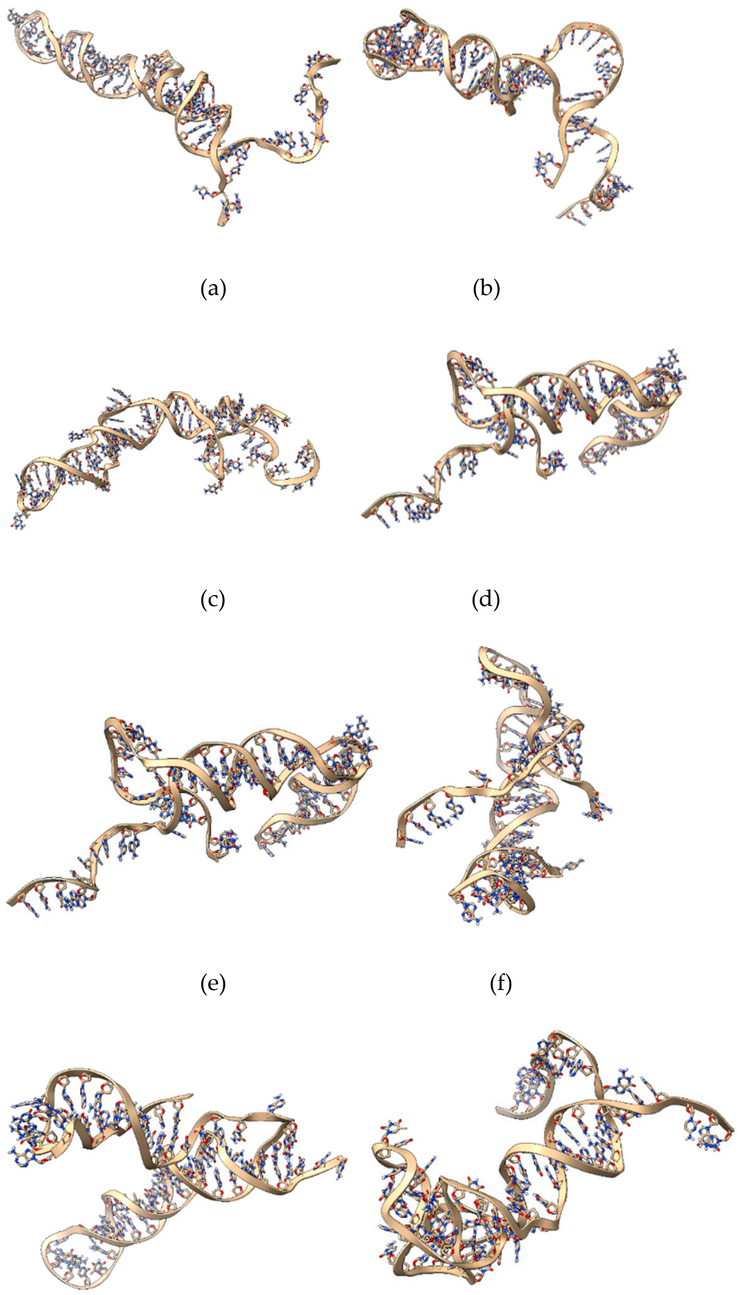

Figure S2. Tertiary structure of (a) Sequence 1; (b) Sequence 2; (c) Sequence 3; (d) Sequence 4; (e) Sequence 5; (f) Sequence 6; (g) Sequence 7 and (h) Sequence 8. With 80nt.

The tertiary structure of aptamers with 40 nt are presented in fifure S3

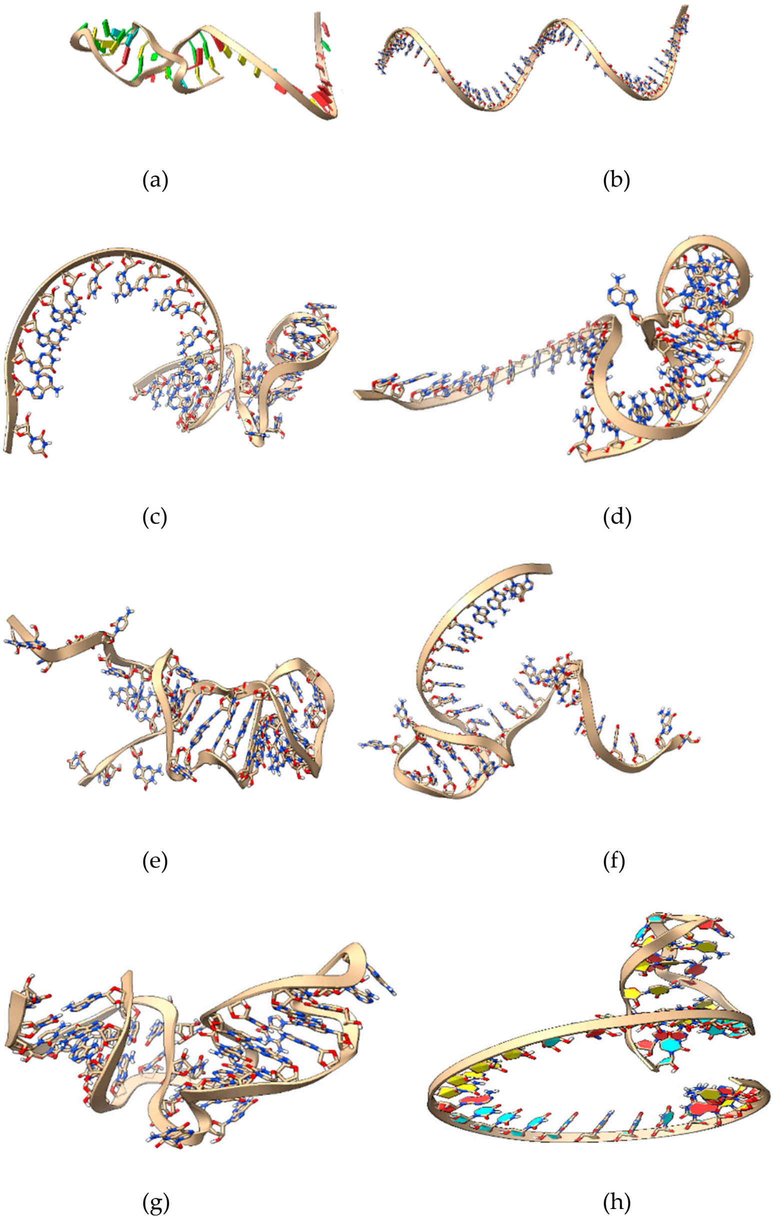

Figure S3. Tertiary structure of: aptamerSEQ1, (a); aptamerSEQ2, (b); aptamerSEQ3, (c); aptamerSEQ4, (d); aptamerSEQ5 (e); aptamerSEQ6, (f) and aptamerSEQ8, (h). All with sequences of 40nt.

The Docking scores for the 8 aptamers are presntedin Table S1

Table S1. Docking scores of the eight aptamerSeqs

Aptamer	Docking Score	Confidence Score	Ligand RMSD
1	-209.87	0.8999	134.62
2	-267.41	0.8754	145.31
3	-346.78	0.9562	87.32
4	-398.45	0.94.12	82.45
5	-227.12	0.9023	167.76
6	-301.45	0.8834	189.43
7	189.76	0.9167	243.51
8	259.66	0.7845	342.98

Docking scores of the Negative Control of protein chCD40 are presented in Table S2

Table S2. Docking Scores of Protein Negative Control of protein CD40

Complex Apt-Prot	Seq3-6PE8	Seq4-6PE8	Seq3-4GIQ	Seq4-4GIQ	Seq3-AF AO Seq4-AF AO A803Y327 A803Y327
Docking Score	-420.17	-459.48	-357.47	-357.47	-383.58 -330.36
Confidence Score	0.9955	0.9955	0.9801	0.9759	0.9907 0.9736
Ligand RMSD	157.15	138.46	212.57	97.34	111.04 218.26

As an example of the PLIP detected interaction information, the residue number, the AA simbol, distance, donor atom number and acceptor atom number are presented in Table S3 only for the H bonding.

Table S3. H bonding of Seq3Aptamer with CD40 residues, PLIP.

Residue	AA	Distance	Donor atom	Aceptor Atom	NT

391	Asn	2.55	236	3241 O3	
421	Gln	2.83	285	3187 O3	
551	Glu	3.60	3265	467 O3	
701	Ser	3.76	2481	679 O3	
701	Ser	3.95	2483	679 O3	
1011	Thr	3.21	3134	1154 O2	
1131	Asp	3.46	1321	3015 O3	
1131	Asp	3.46	3015	1321 O3	
1141	Ala	3.46	1327	3015 O3	
1151	Ser	1.97	1342	3078 O3	
1231	Glu	3.41	3035	2677 O2	
1491	Glu	2.79	1804	2698 O3	
1811	Asn	3.10	2286	1452 O2	
1811	Asn	2.65	2293	2677 O2	

As example of the Salt Bridges interaction, the residue number the distance , Ligand group and the number of the ligand from PLIP are presented in Table S4.

Table S4. Salt Bridges of Seq3Aptamer with CD40 residues, PLIP.

INDEX	Residue	Amino Acid	Distance	Ligand group	Ligand Atoms
1	691	His	5.37	Phosphate	3118, 3118, 3117, 3121, 3119, 3120
2	991	His	4.44	Phosphate	3140, 3140, 3142, 3139, 3141, 3143
3	1981	Lys	3.14	Phosphate	2718, 2718, 2719, 2721, 2717,2780
4	1781	Lys	5.06	Phosphate	2697,2697, 2698,2700,2696, 2699
